# People, organizational, and leadership factors impacting informatics support for clinical and translational research

**DOI:** 10.1186/1472-6947-13-20

**Published:** 2013-02-06

**Authors:** Philip RO Payne, Taylor R Pressler, Indra Neil Sarkar, Yves Lussier

**Affiliations:** 1 Department of Biomedical Informatics, The Ohio State University, Columbus, OH, USA; 2 Department of Computer Science, Department of Microbiology and Molecular Genetics, University of Vermont, Burlington, VT, USA; 3 Department of Medicine and Engineering, University of Chicago, Chicago, IL, USA

## Abstract

**Background:**

In recent years, there have been numerous initiatives undertaken to describe critical information needs related to the collection, management, analysis, and dissemination of data in support of biomedical research (J Investig Med 54:327-333, 2006); (J Am Med Inform Assoc 16:316–327, 2009); (Physiol Genomics 39:131-140, 2009); (J Am Med Inform Assoc 18:354–357, 2011). A common theme spanning such reports has been the importance of understanding and optimizing people, organizational, and leadership factors in order to achieve the promise of efficient and timely research (J Am Med Inform Assoc 15:283–289, 2008). With the emergence of clinical and translational science (CTS) as a national priority in the United States, and the corresponding growth in the scale and scope of CTS research programs, the acuity of such information needs continues to increase (JAMA 289:1278–1287, 2003); (N Engl J Med 353:1621–1623, 2005); (Sci Transl Med 3:90, 2011). At the same time, systematic evaluations of optimal people, organizational, and leadership factors that influence the provision of data, information, and knowledge management technologies and methods are notably lacking.

**Methods:**

In response to the preceding gap in knowledge, we have conducted both: 1) a structured survey of domain experts at Academic Health Centers (AHCs); and 2) a subsequent thematic analysis of public-domain documentation provided by those same organizations. The results of these approaches were then used to identify critical factors that may influence access to informatics expertise and resources relevant to the CTS domain.

**Results:**

A total of 31 domain experts, spanning the Biomedical Informatics (BMI), Computer Science (CS), Information Science (IS), and Information Technology (IT) disciplines participated in a structured surveyprocess. At a high level, respondents identified notable differences in theaccess to BMI, CS, and IT expertise and services depending on the establishment of a formal BMI academic unit and the perceived relationship between BMI, CS, IS, and IT leaders. Subsequent thematic analysis of the aforementioned public domain documents demonstrated a discordance between perceived and reported integration across and between BMI, CS, IS, and IT programs and leaders with relevance to the CTS domain.

**Conclusion:**

Differences in people, organization, and leadership factors do influence the effectiveness of CTS programs, particularly with regard to the ability to access and leverage BMI, CS, IS, and IT expertise and resources. Based on this finding, we believe that the development of a better understanding of how optimal BMI, CS, IS, and IT organizational structures and leadership models are designed and implemented is critical to both the advancement of CTS and ultimately, to improvements in the quality, safety, and effectiveness of healthcare.

## Introduction

In recent years, there have been numerous initiatives undertaken by members of the biomedical informatics (BMI) community to describe people, organizational, and leadership factors that influence the collection, management, analysis, and dissemination of data, information, and knowledge in support of biomedical research [1-4]. A common theme spanning these reports has been the critical role of predisposing or enabling factors that may impact the likelihood of achieving the promise of computational-approaches to such information needs [5]. As clinical and translational science (CTS) emerges as a national priority, the correlating growth in the scale and scope of CTS research programs also causes a corresponding increase in the acuity of such information needs. [6-8]. At the same time, systematic evaluations of optimal people, organization, and leadership models related to the provision of data, information, and knowledge management technologies and methods relevant to the conduct of CTS are notably lacking. In this report, we describe an effort, focused on such factors in the specific context of clinical and translational science programs situated in academic healthcare centers (AHCs), intended to address the preceding gap in knowledge.

## Background

### Key terms and definitions

**Figure 1:**
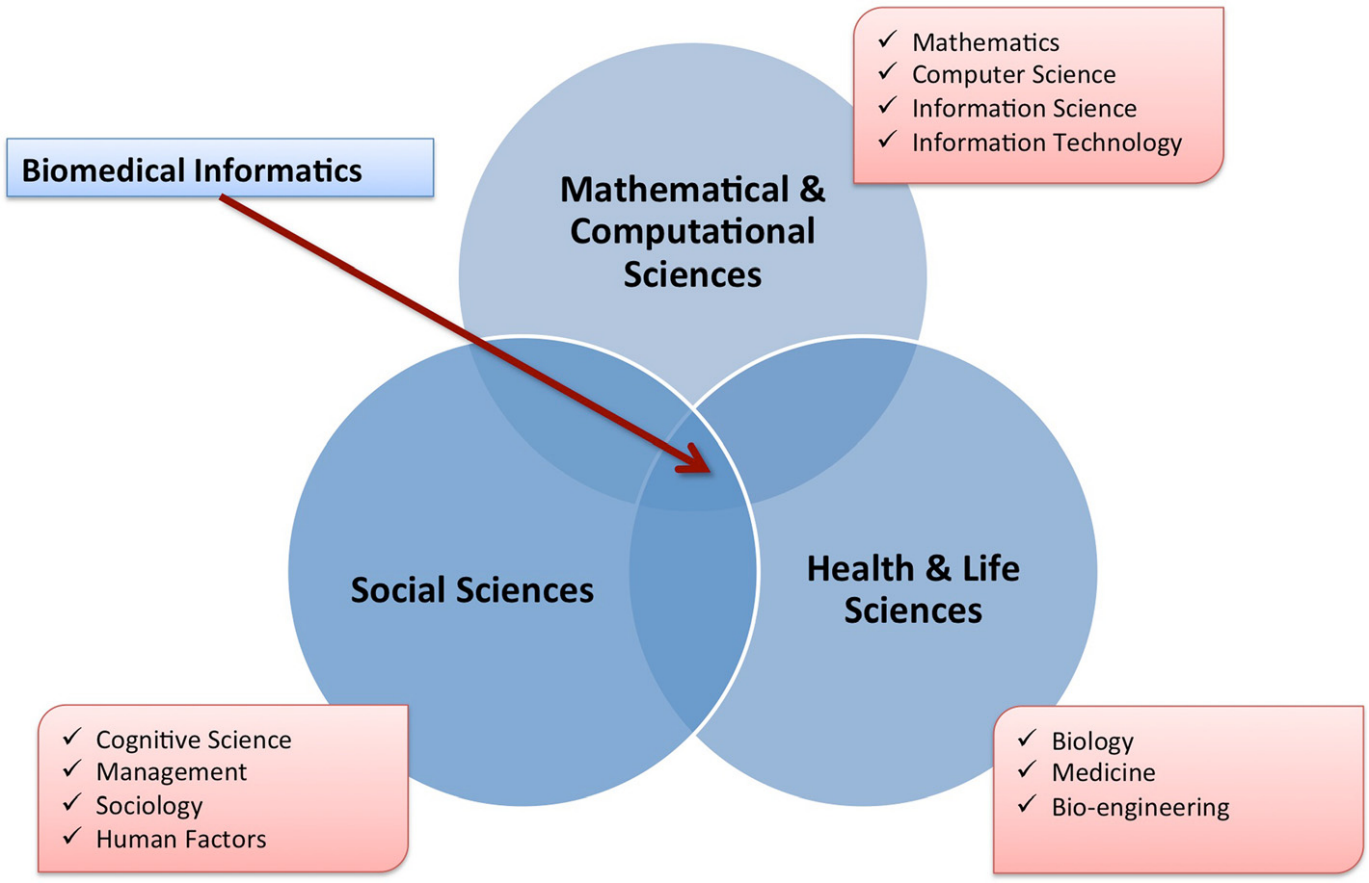
The field of biomedical informatics represents an intersection between the areas of mathematics and computational science, social sciences, and health and life sciences.

Given that the intent of this report is to describe a survey and evaluation of such people, organizational and leadership factors that impact informatics support for CTS, it is important to provide shared context for key terms and concepts that we will use in the remainder of the manuscript. Do address this nee, we will utilize the following working definitions:

• *Computational and Information Science*: The term Computer Science (CS) came into common use in the 1960’s, but does not necessarily correlate with a specific and community accepted definition of the focus and scope of the field. In a broad sense, CS can be defined, according to the Association for Computing Machinery (ACM) conventions, as the branch of science concerned with the theoretical and applied use of computers to process information. Of note, significant debate exists with regards to whether CS is a form of applied mathematics, engineering, or a distinct discipline unto itself [9]. In a similar manner, a broadly accepted definition for the domain of Information Science (IS) is also lacking. Per an assessment of the relationships between CS, IS, and Biomedical Informatics by Shortliffe and Blois, the label, IS, *“is occasionally used in conjunction with computer science, originating in the field of library science and is used to refer, somewhat generally, to the broad range of issues related to the management of both paper-based and electronically stored information”*[10].

• *Biomedical Informatics*: Biomedical Informatics (BMI) is “the interdisciplinary field that studies and pursues the effective uses of biomedical data, information, and knowledge for scientific inquiry, problem solving, and decision making, motivated by efforts to improve human health” [11]. A visualization of the core scientific domains that contribute to the basic and applied practice of BMI is found in Figure 1.

• *Information Technology:* The field of Information Technology (IT) is concerned with the application of various forms of technology and correlative data, information, and knowledge-centric processes in order to achieve outcomes dictated by operational and business needs or goals [12].

In addition to the preceding working definitions, where applicable, we utilize the National Institutes of Health (NIH) definitions [13] for both clinical and translational research, as follows:

• *Clinical Research:* Research with human subjects that is:

Patient-oriented research. Research conducted with human subjects (or on material of human origin such as tissues, specimens, and cognitive phenomena) for which an investigator (or colleague) directly interacts with human subjects. Excluded from this definition are in vitro studies that utilize human tissues that cannot be linked to a living individual. It includes:

• Mechanisms of human disease

• Therapeutic interventions

• Clinical trials

• Development of new technologies

• Epidemiological and behavioral studies

• Outcomes research and health services research

• *Translational Research:* Translational research includes two areas of translation. One is the process of applying discoveries generated during research in the laboratory, and in preclinical studies, to the development of trials and studies in humans. The second area of translation concerns research aimed at enhancing the adoption of best practices in the community. Cost-effectiveness of prevention and treatment strategies is also an important part of translational science.

It is important to note that, like all such taxonomic schemas, these working definitions are neither exclusive nor exhaustive; rather, they are intended to be exemplary in the absence of widely adopted standard conceptual and operational definitions of the involved domains. The authors fully anticipate a variety of exceptions to such a rubric, but also believe that these working definitions provide a sufficiently broad coverage of the targeted domains so as to provide readers with a broad understanding of their similarities and differences.

### Contributing prior work and motivation

As has been noted in numerous recent reports, the modern academic biomedical research environment has experienced a fundamental shift towards the conduct of transdiciplinary and integrative basic, clinical, and translational research, commonly referred to as clinical and translational science (CTS) [7,8]. Such an approach to biomedical innovation stands in stark contrast to historical models involving investigators operating in domain specific “silos”. Such transdiciplinary research programs are intended to achieve both economies of scale and systems-level impact that are not attainable in single investigator studies and laboratories [2,6,14,15]. A common infrastructural basis for such activities is the use of computational science, information science, biomedical informatics, and information technology derived methods and tools (e.g., to support team collaboration, project planning, data management, knowledge generation, and results dissemination to name a few of many such applications) [1,14]. This complex set of information needs requires the creation of optimized organizational structures and the engagement and leadership of appropriately trained individuals in order to support the systematic delivery of BMI expertise and resources [16-19]. It has been argued that such leaders and structures are often uniquely positioned to facilitate the coordination and harmonization of the relevant scientific and technical domains required to address the core information needs commonly found in the modern academic CTS enterprise [3,6,14,20]. Unfortunately, despite the potential benefits afforded by the use of computational science, information science, biomedical informatics, and information technology derived methods and tools to support CTS, there are also numerous reports that demonstrate that achieving such benefits in CTS environment is extremely challenging. Such difficulties are often attributable to a combination of one or more factors, including: (1) a lack of understanding by key decision makers as to the synergies and distinctions between the aforementioned fields [21]; (2) the absence of widely reported and easily replicable organizational models capable of supporting integrative BMI programs in the academic CTS enterprise; and, (3) the inconsistent engagement or availability of individuals with appropriate skills and training to lead such efforts [22].

## Methods

Motivate by the issues identified in the preceding disucssion, and in order to study the important factors that serve to influence access to informatics expertise and resources relevant to the CTS domain at AHCs, we conducted a mixed-method study involving two sequential phases: 1) we surveyed a convenience sample of subject matter experts (SMEs) associated with CTS-focused informatics programs at AHCs; and 2) we triangulated and contextualized the results of this survey through a grounded-theory based thematic analysis [23-25] of publically available documents (such as annual reports, web sites, and published literature) that served to describe the integration and/or activities of relevant BMI, CS, IS, and IT programs and leaders to the CTS domain at those institutions. The specific methods associated with these phases are described below:

### Phase one: structured survey

An anonymous electronic survey request was sent via email to a convenience sample of SMEs, who are both affiliated with the national Clinical and Translational Science Award (CTSA) Informatics Key Functional Committee (IKFC), and are members of the American Medical Informatics Association (AMIA) Clinical Research Informatics working group (CRI-WG). Of note, these two groups of potential participants are not mutually exclusive; however, potential respondents were requested to complete the survey only once. The indicated survey instrument, implemented using REDCap Survey [26], was designed via an interative and heuristic process involving the authors of this manuscript (PROP, TRP, INS, YL). This survey employed a combination of Likert-scale and free-text responses, and asked respondents to rate various aspects of the research environment at their respective institution, including: (1) access to services and expertise of CS, IS, BMI, and IT; (2) the coordination of CS, IS, BMI, and IT leadership at their institution; and, (3) the funding model used for CS, IS, BMI, and IT services relative to research missions. Additionally, respondents were asked to characterize the relationship of the leaders of CS, IS, BMI, and IT at their institution. Further details concerning the specific survey questions utilized in this study can be found in Tables 1, 2, 3, and Additional file 1. This survey instrument was subject to face-validity checking by a convenience sample of potential respondents affiliated with the CTSA-funded Center for Clinical and Translational Science (CCTS) at The Ohio State University, prior to its distribution. This research protocol was reviewed and approved by the Institutional Review Board of The Ohio State Univeristy. 

**Table 1:** Summary of questions and responses from electronic survey instrument

**Question**	**Responses**
*1. Access to CS/IS/IT services and expertise*	22.5% Very good (7)
	22.5% Good (7)
	**45.1% Fair (14)**
	3.2% Poor (1)
	No answer provided (1)
*2. Access to BMI services and expertise*	16.1% Very good (5)
	**32.3% Good (10)**
	**35.5% Fair (11)**
	12.9% Poor (4)
	3.2% No BMI services (1)
*3. Coordination of BMI-CS/IS/IT leadership*	25.8% Very good (8)
	12.9% Good (4)
	**32.3% Fair (10)**
	25.8% Poor (8)
	3.2% No BMI services
*4. Description of CS/IS/IT and BMI leader’s relationship*	32.3% Integrated/coordinated (10)
	**48.3% Not integrated/coordinated (15)**
	19.3% Other (6)
*5. Funding of CS/IS/IT and/or BMI services for research*	6.4% Very good
	16.1% Good (5)
	**48.3% Fair (15)**
	22.5% Poor (7)
	6.4% Other (2)

Ratings that received a plurality of responses are indicated in bold.

**Table 2:** Survey responses stratified by the presence of a formal BMI academic unit

		**BMI academic unit**	**No BMI unit**
**Access to BMI services and expertise**	*Very Good/Good*	60%	27%
	*Fair/Poor*	40%	63%
**Access to CS/IS/IT services and expertise**	*Very Good/Good*	60%	9%
	*Fair/Poor*	35%	72%
**Coordination of CS/IS/IT/BMI Leadership**	*Very Good/Good*	60%	0%
	*Fair/Poor*	40%	90%

**Table 3:** Survey responses stratified based upon the description of the relationship between BMI and IT leaders

		**Coordinated and integrated**	**Not coordinated or integrated**
**Access to IT services and expertise**	*Very Good/Good*	80%	26%
	*Fair/Poor*	20%	73%

### Phase two: triangulation and contextualization via thematic analyses

In order to provide further context surrounding the preceding survey results, we performed a qualitative analysis of self-reported documentation concerning the structure and function of several AHCs with large-scale CTS research programs (e.g., via web-based content, self-published reports, and peer-reviewed literature). These materials were identified via a systematic search process that included:

1) A web-search, conducted using the Google search engine, employing the targeted institutions/organizations name and the free text phrases: [“Informatics”, “Information Technology”, “Information Science”, “Computation”, “Computing”, “Computer Science”, “Data Management”] AND [“Clinical Science” OR “Translational Science”].

2) A publication-search, conducted using the PubMed portal, employing the targeted institutions’/organizations’ names and the Medical Subject Heading (MeSH) terms: 1) “Information Science” (which subsumes a broad scope of CS, IS, BMI, and IT concepts); 2) “Medical Translational Research”; and, 3) “Clinical Research”.

Of note, with regard to the web-search stragey (i.e. item (1) above), such search results were filtered and sub-selected heuristically by the authors to identify documents or publications that were most likely to contribute to the objectives of this study phase. Examples of the types of documents selected during this process included: 1) informational web pages; 2) annual reports; 3) white papers; 4) news items or press released; and 5) linked publications from journals or conference proceedings. This corpus of documents was then subject to an iterative thematic analysis using a grounded-theory approach, executed in an iterative, team-based manner by two of the authors (PROP, TRP). The results of this analysys were then used to inform a set of high-level observations concerning the people, organizational, and leadership models used to provide access to CS, IS, BMI, and IT services and expertise in support of their CTS mission area(s).

## Results

### Phase one: structured survey

A total of 31 individuals responded to the previously described survey. This group of respondents self-identified themselves as: (1) leaders in BMI domain (n = 23); (2) leaders in CS, IS, or IT (n = 4); (3) BMI researchers/educators (n = 2); (4) CS, IS or IT researcher/educator (n = 1); and, 5) involved in CS or IT development and evaluation activities (n = 1). When asked to describe the institutions at which they work, 64.5% of the respondents reported that their institution had a BMI department or other formal academic unit (35.5% did not). 54.8% of the respondents reported that their institution has a BMI training program (41.9% did not; 1 respondent did not answer this question). While the survey was anonymous, some respondents did choose to identify the institution at which they work. These institutions include:

•Duke University

• Vanderbilt University

• Johns Hopkins University

• Columbia University

• Oregon Health and Science University

• University of Iowa

• University of North Carolina, Chapel Hill

• University of Texas, Galveston

• University of Pittsburgh

Additional questions from and responses to the survey are summarized in Table 1.

When analyzing the results of the survey, the presence of a formal academic unit for BMI and the perceived relationship between CS/IS/IT and BMI leaders reveals distinct differences in the responses for each respondee. Statistical significance could not be achieved, due to the small sample size, but the qualitative features of the data are very informative. Respondent who are affiliated with an institution that has a formal academic unit for BMI were more likely to describe their access to BMI and CS/IS/IT services as “Very Good” or “Good” and were more likely to describe the coordination between CS/IS/IT and BMI leaders as “Very Good” or “Good” than those whose institutions did not have a formal BMI unit. These results are summarized in Table 2.

In addition, those respondents who described the relationship between CS/IS/IT and BMI leaders as coordinated and integrated appeared to be more likely to perceive higher access to IT services and the overall coordination of BMI and IT leaders than those who characterized the relationship as not coordinated, as shown in Table 3. However, it is important to note that these data do not suggest that there is a correlation between an institution having a BMI academic unit and a perception of a coordinated/integrated relationship between BMI and IT leaders, based on a risk ratio of 0.61 (0.08, 1.06).

### Phase two: triangulation and contextualization via thematic analyses

To provide further context surrounding the preceding survey results, we also undertook the previously described thematic analysis of self-reported materials describing BMI and CS/IT related organizational structure and function of several academic health centers (AHCs) with large-scale transdiciplinary research programs. Table 4 provides a summary of the document corpora retrieved for this phase. The results for the grounded-theory thematic analysis, conducted by two of the authors (PROP, TRP), identified five major themes that served to predispose or enable the effective coordination of CTS relevant BMI and CS/IT expertise and resources, as follows:

1) The nature of the leadership model via which decision makers in the BMI and CS/IT domains coordinated their activities;

2) The nature of the funding model that was used to support both BMI and CS/IT personnel and resources targeting CTS information needs;

3) The presence of formal mechanisms for the facilitation of research to production translation in the context of software products supporting CTS;

4) The availability of BMI-focused and CTS-relevant training program(s); and

5) The presence of a formal BMI home at the given institution.

**Table 4:** Scale and scope of document corpora retrieved for the purposes of thematic analyses

**Institution**	Number of documents retrieved/selected via web search*	**Number of documents retrieved/selected via publication search**
Duke University	1/0	3/3
Vanderbilt University	6/6	4/4
Johns Hopkins University	3/1	8/8
Columbia University	5/4	6/6
Oregon Health and Science University	3/2	2/2
University of Iowa	3/2	1/1
University of North Carolina, Chapel Hill	3/2	5/5
University of Texas, Galveston	0/0	0/0
University of Pittsburgh	6/5	11/11

* Documents identified as either being a CV/resume or a peer-reviewed publication were censored from analysis relative to the web search strategy used during this study phase.

For the purposes of this evaluation, search results were initially limited to documents published within the last three years (2009-2012).

As Table 5 demonstrates, examples were drawn from three exemplary cases, sub-selected from our overall group of targeed institutions evaluated during this phase, on the types of organizational features that corresponded with the five aforementioned themes.

**Table 5:** Defining characteristics of academic health centers (AHCs) that have successfully integrated computational science, biomedical informatics, and information technology mission areas and leadership models in order to advance research and healthcare delivery

**Exemplary case**	**Axis 1: leadership model**	**Axis 2: funding model**	**Axis 3: research to production translation**	**Axis 4: training program(s)**	**Axis 5: biomedical informatics home”**
**Case 1:***Mid-sized Public University*	Coordinated^1^	Academic and Health System Support	Comprehensive^3^	Professional^5^ and Research^6^ Oriented	Academic Department
**Case 2:***Large Public University*	Integrated^2^	Academic and Health System Support	Minimal^4^	Research^6^ Oriented	Center
**Case 3:***Large Private University*	Integrated^2^	Academic and Health System Support	Comprehensive^3^	Professional^5^ and Research^6^ Oriented	Academic Department

^1^ Biomedical Informatics leaders advise IT leaders concerning AHC IT strategy and services.

^2^ Biomedical Informatics and IT leaders jointly oversee AHC IT strategy and services.

^3^ Widespread deployments of production technologies (i.e., EHR platforms) derived from BMI-driven research and development programs.

^4^ Limited and/or small-scale deployments of production technologies (i.e., research-specific data management systems) derived from BMI-driven research and development programs.

^5^ Terminal masters and certificate programs, usually focusing on the application of informatics theories and methods.

^6^ Terminal masters and doctoral programs, as well as post-doctoral fellowships, usually focusing on the discovery and validation of novel informatics theories and methods.

These cases have been rendered anonymous in order to reduce potential sources of bias. For the purposes of this table: 1) “IT Leadership Model” refers to the type of relationship between CS/IS/IT leaders and BMI leaders at the organization relative to operational decision making; 2) “Funding Model” refers to the sources of funding used to support research-specific CS/IT/IT and BMI resources; 3) “Research to Production Translation” refers to the degree to which software products generated during the course of CS/IS and/or BMI research are translated into production platforms/tools; 4) “Training Program(s)” refers to the types of BMI training available at the institution; and 5) “Biomedical Informatics Home” indicates what type of organizational unit houses BMI at the given institution.

## Discussion

In the following sub-sections, we will: 1) review the implications of the findings generated during the course of this study; and, 2) present a series of perspectives, informed by the preceding findings, concerning important strategies that can optimize access to CS/IS/IT and BMI expertise and services in the modern academic CTS enterprise. We will also briefly discuss the limitations of our study, as well as a series of future research directions based upon the outcomes of this body of work.

### Implications of study findings

When interpreting the results of our structured survey and thematic analyses, a number of interesting and notable findings arose, including:

• In the case of the three survey indicators of the ability of researchers to access critical data, information, and knowledge management expertise and resources, most respondents described such access being “Fair”, with a minority of individuals describing such access as being “Good” or “Very Good”. These results would appear to indicate that such organizational characteristics remain an area of concern and may in fact serve as impediments to the conduct of CTS, despite the previously described body of evidence indicating the importance of such infrastructure and personnel.

• Similarly, in the case of the two survey indicators of people or leadership issues surrounding the coordination of leadership related to CS, IS, BMI, and IT, again, most respondents indicated that such factors were either “Fair” or “Not intergated/coordinated”. This would appear to indicate characteristics of concern relative to the conduct of efficient and timely CTS programs.

• In terms of the ability to fund criticial CS, IS, BMI, and IT resources and services, the preponderance of responses indicate that such funding was either “Fair” or “Poor” – an outcome that may be explantatory for the two preceding points, and again, one that would appear to indicate characteristics of concern relative to the conduct of efficient and timely CTS programs.

• As was introduced earlier, survey respondents at organizations that housed a formal academic unit for BMI were more likely to describe their access to BMI and CS/IS/IT services as “Very Good” or “Good” and were more likely to describe the coordination between CS/IS/IT and BMI leaders as “Very Good” or “Good” when compared to respondents at organizations that did not have a formal BMI unit. This would appear to show that formal academic BMI units are extremely important relative to supporting and enabling access to CS/IS/IT and BMI expertise and resources in the modern academic CTS enterprise.

• Finally, when considering the preceding outcomes, in light of the results of our subsequent thematic analyses of available public-access materials describing the CS/IS/IT and BMI components of major academic CTS enterprises, there is great discordance between such reports and the perceptions of scientific and operational leaders at those same institutions. This is an area of concern, as it may indicate that an understanding of these types of critical people, organizational, and leadership issues may not be consistent or shared across all levels of CTS-focused institutions. This issue may lead to a negative influence on important decision-making concerning the leadership, resourcing, and provision of such mission critical services, personnel, and infrastructure.

When taken as a whole, the preceding impressions and outcomes related to our study findings would appear to indicate that major people, organizational, and leadership issues may be impeding access to mission crticial CS/IS/IT and BMI resources and expertise in modern academic CTS enterprises, including, but not limited to, the engagement and coordination of appropriately trained leaders; the provision of sufficient resources to support and sustain CTS-focused CS/IS/IT and BMI resources and personnel; andm a lack of understanding of the preceding issues at all pertinent organizational levels.

### Author perspectives concerning important strategies that can optimize access to biomedical informatics expertise and services in the modern academic CTS enterprise

**Figure 2:**
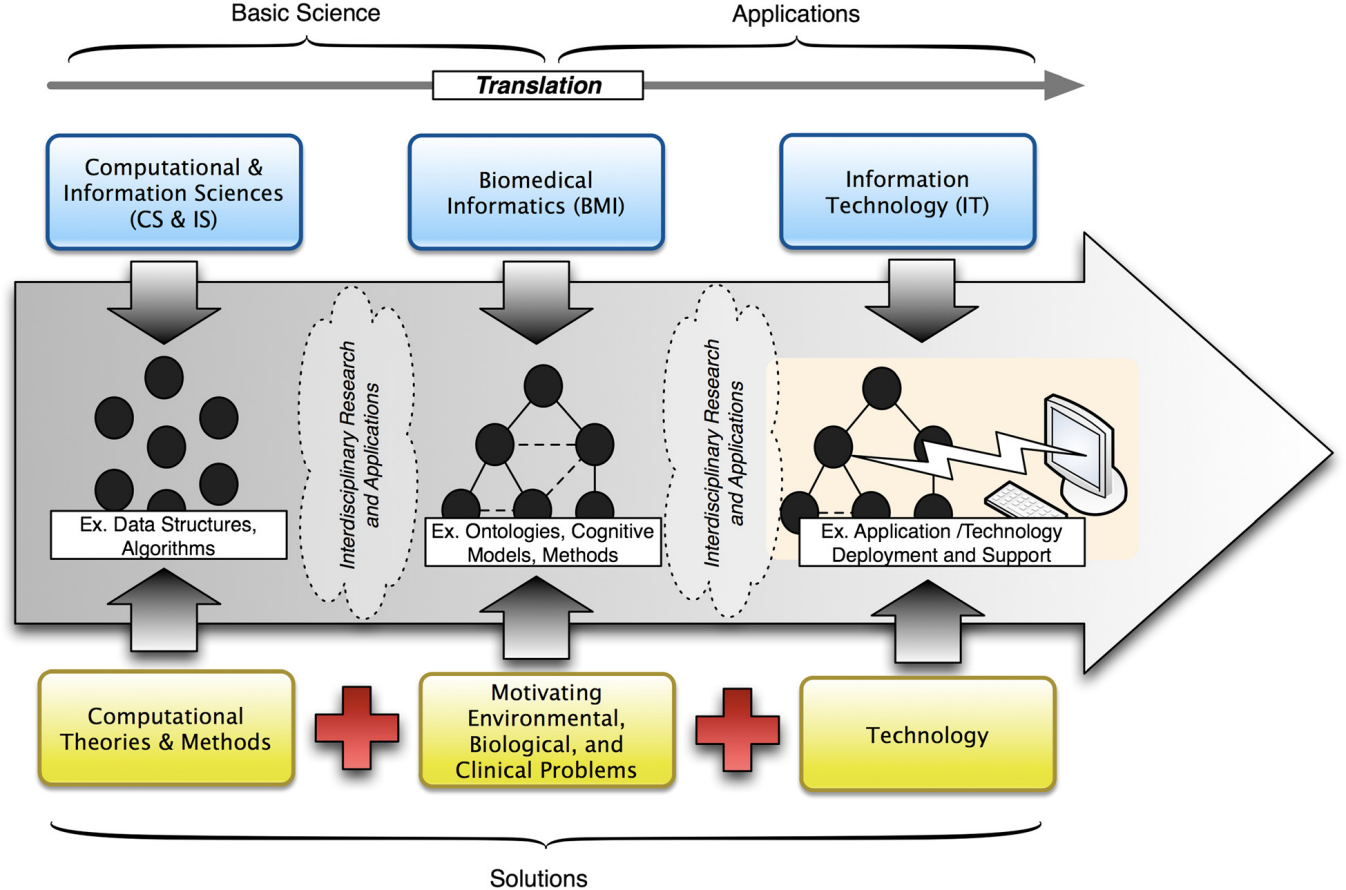
**When considering the translational continuum between basic science and applications, biomedical informatics involves the use of both the computational theories and methods and information technology in order to generate meaningful solutions and results.** The intersection points between component disciplines that comprise this translational spectrum incorporate “fuzzy” boundaries between disciplines, wherein interdisciplinary investigators engage in both research and application development that incorporate aspects of complementary theories and methods.

Based upon the preceding study findings, we have developed two high-level perspectives that relate to the critical people, organizational, and leadership factors that influence and/or predispose access to CS/IS/IT and BMI expertise and services in academic CTS settings. These perspectives are summarized below:

1) It is critical to understand the differences and opportunities for harmonization between CS, IS, BMI, and IT as they related to the operations of the modern academic CTS enterprise: As stated in the introduction, and as has been described in the published literature, CS, IS, BMI, and IT have remarkably complementary and synergistic roles in the modern research enterprise [21,22]. Given the need for comprehensive, resource-efficient, and efficacious data, information, and knowledge management in the majority of basic, clinical, and translational research programs, investigators and their collaborators increasingly expect that CS/IS/IT and BMI methods and platforms, intended for use in such capacities, are made accessible to them in a high-availability manner. Furthermore, such end-users expect CS/IS/IT and BMI methods and platforms to be accessible with minimal barriers for adoption or adaptation [2,3,6,14,27,28]. The provision of such resources and technologies requires a number of approaches, including: (1) the rapid development, validation, and translation to production deployment of innovative computational and informatics derived tools and methods; (2) the ongoing support and management of core research IT infrastructure; and, (3) the derivation and execution of comprehensive strategies surrounding the two aforementioned areas. As illustrated in Figure 2, the ability to achieve such a vision requires a combination of solutions, incorporating computational and information science theories and methods, which are contextualized for the biomedical domain using BMI theories and methods, coupled with motivating biological, and clinical problems that are realized via supporting information technology platforms. At each step in such a continuum, there is a corresponding role for professional leadership and guidance relating to multiple domains. For example, individuals who engage in CS/IS research are ideally positioned to define and validate novel theories and methods that can support the management and manipulation of data generated throughout all aspects of the research enterprise. Similarly, individuals with BMI competencies are specifically trained to map these theories and methods, for the motivating environmental, biological, and clinical problems, and design methodological and strategic approaches, to such combinatorial solutions. Finally, IT professionals are appropriately trained and have access to information systems deployment, support, and management methods and physical computing resources such that the products generated by BMI professional can be “hardened” and made available to end-users in a high-availability manner. Spanning such a model is what can be thought of as an *informatics translational cycle*, in which basic science discoveries (e.g., computational theories and methods, and their efficacious mapping and alignment with motivating problem spaces) can be translated into applications, such as end-user accessible information systems. Much as is the case with the prevailing understanding of the clinical and translational research paradigm, such an informatics translational cycle, by necessity, involves the collaboration of a multi-disciplinary team with key leaders, whom possess expertise and competencies in all contributing domains [3,7,21,29].

2) The identification and engagement of appropriately trained and empowered CS, IS, BMI, and IT leaders, with effective relationships between such individuals, is of the utmost importance to the creation and maintenance of an effective CTS environment: Building upon the analysis of the relationships between CS, IS, BMI, and IT in the modern academic CTS environment, we have identified a number of critical strategies that are essential to the successful leadership and organizational delivery of BMI, CS, and IT expertise and resources needed to support clinical and translational research. These strategies are summarized below:

a. *It is critical for organizations to understand and address the differences between leadership needs in CS, BMI, and IT.* For example, IT leaders generally do not possess the training and core competencies necessary to lead BMI focused efforts (such as the alignment of CS/IS theories and methods with driving basic science, clinical, and translational research problems), just as BMI leaders generally do not possess the training and core competencies to manage and oversee production IT deployment environments and support services. As a result, it is neither appropriate nor efficacious for IT leaders to oversee BMI related strategies and initiatives or vice-versa. The results of the survey indicate that when there is a formal BMI academic unit in place, there is a distinct difference in the perceived access to both BMI and IT services and expertise as well as a perceived coordination between BMI and IT leaders.

b. *Partnerships between CS, IS, BMI, and IT organizations or structures and leaders must be collaborative, balanced, and durable.* In many organizations, these types of relationships are either dysfunctional or even adversarial [14]. Where successful partnerships do exist, they often rely upon the personalities of the leaders involved, rather than formal structures and agreements. This is further evidenced in our survey data, where a formal BMI academic unit does not affect the perceived level of coordination and integration between BMI and IT leaders. Given the need to leverage collaborations between these domains, in order to realize economies of scale and address end-user information needs, they must be formalized in a balanced manner and established within organizational governance models.

c. *The provision of research computing, informatics, and IT infrastructure must be valued and supported in a manner commensurate with clinical and operational resources.* Presently, in the vast majority of AHCs, research computing is addressed in silos or at the individual investigator level, with little or no widespread institutional support [14]. This lack of resourcing and evaluation for research computing is based on a traditional, but we believe indefensible, position that research is a distinct enterprise from clinical care and overall organizational operations. Given the increasing demand for personalized healthcare, combined with cost controls and an increased focus on re-engineering healthcare delivery to enable quality and outcomes improvement, such a distinction directly conflicts with the goals we are pursuing at a national level. Furthermore, the previously described *informatics translational cycle* is unlikely to be successful in the absence of the elimination of such barriers and the appropriate institutional support of research computing.

d. *Informatics workforce development is a central and supporting endeavor in well-integrated environments.* The ability to recruit, initially train, and facilitate the ongoing knowledge-base development of informatics researchers and professionals is of the utmost importance in terms of realizing the benefits of the previously defined *informatics translational cycle.* The ability to achieve such capabilities is almost always highly reliant on the establishment and support of an appropriate academic “home” for informatics, as well as the ongoing operation of a comprehensive suite of training programs at multiple levels.

### Limitations of our findings and future directions

While we believe that the study and findings described in this report are broadly generalizable and extensible, there are a number of important limitations that should be noted, including:

• The relatively small, convenience sample of respondents engaged in the electronic survey of clinical and translational research thought leaders, as well as the potential for a self-selection bias relative to survey participation;

• The inclusion of only individuals with self-described expertise in CS, IS, BMI, or IT in the survey process, thus omitting the perspective of leaders in the CTS domain with other backgrounds or expertise.

• The use of thematic and grounded-theory analyses to generate some of our findings, relying on a small number of subject-matter experts to execute such methodologies; and,

• The reliance upon descriptive statistics to support our findings, as a result of the two preceding limitations.

In response to these limitations, we intend to conduct a broader survey as part of future work, as well as engage a larger group of subject matter experts in a structured focus group and interview process.

Furthermore, and in addition to the aforementioned limitations, there are two additional classes of factors that were raised during our analysis, (particularly during Phase 2) that may influence or otherwise impact the availability of and access to CS, IS, BMI, and IT in the modern academic CTS environment, namely: 1) the differential financial models used to fund and support such activities; and 2) the process by which investigational software platforms or tools generated in CS, IS, or BMI laboratories are “translated” into operational IT systems. However, the specific features and details that may serve to explain these factors extend beyond the scope of the study design and data capture used in this particular project, and thus represent opportunities for future lines of research.

## Conclusions

As opposed to traditional investigator and domain specific approaches to driving biological, clinical, and translational problems, the modern academic CTS environment has experienced a major paradigm change that demands the increasing conduct of transdiciplinary and integrative basic, clinical, and translational research. Central to the ability to form, support, and realize the benefits of such transdiciplinary and team science is the appropriate use of CS, IS, BMI, and IT methods and tools. However, the ability to achieve these benefits requires the engagement and support of leaders in all three domains, working in a balanced, well supported, and synergistic manner. Unfortunately, such people, organizational, and leadership models remain difficult to achieve in many large AHCs and similar research organizations for a variety of reasons as introduced and elaborated upon in this report. In light of these challenges, we offer a set of high-level strategies intended to inform potential solutions to such issues, which include: 1) the importance of establishing and maintaining organizational constructs that are cognizant of and harmonize between the critical competencies associated with CS, IS, BMI, and IT as those areas pertain to CTS (as opposed to conflating their purposes and capabilities); and, 2) engaging and supporting leaders with appropriate BMI training to ensure that the preceding organizational constructs are appropriately situated and operated (again, as opposed to engaging individuals with general CS, IS, or IT leadership competencies in such positions). It is our intent in doing so to both assist leaders at all levels to critically evaluate such challenges and to catalyze a vigorous community dialogue on this important topic.

## Abbreviations

CTS: Clinical and translational science; AHC: Academic health centers; BMI: Biomedical informatics; CS: Computer science; IS: Information science; IT: Information technology; CTSA IKFC: Clinical and Translational Science Award Informatics Key Functional Committee; AMIA: American Medical Informatics Association; CRI-WG: Clinical research informatics working group.

## Competing interests

The authors of this paper do not have any competing interests to report.

## Authors’ contributions

PROP, INS, and YL contributed to the conceptualization and planning of the study described in this manuscript. PROP, TRP, INS, and YL designed all study data capture instruments. PROP and TRP collected and analyzed all study data. PROP, TRP, INS, and YL participated in the preparation of the manuscript. All authors read and approved the final manuscript.

## Pre-publication history

The pre-publication history for this paper can be accessed here:

http://www.biomedcentral.com/1472-6947/13/20/prepub

## Supplementary Material

Additional file 1 Supplemental file: Electronic Survey Questions. Click here for file
