# Supplementary material for: People, organizational, and leadership factors impacting informatics support for clinical and translational research
Source: BMC Med Inform Decis Mak. 2013 Feb 6;13:20. doi: 10.1186/1472-6947-13-20 (PMC3577661; doi:10.1186/1472-6947-13-20)
Supplement: Additional file 1 — Supplemental file: Electronic Survey Questions. [file 1472-6947-13-20-S1.doc]

**Supplemental File: Electronic Survey Questions**

| **Question** | **Responses** |
| --- | --- |
| 1. What is your primary role at your institution? | - IT leader - IT staff - BMI leader - BMI researcher/educator - Computer or Information Science leader - Computer of Information Science researcher/educator - Other (please describe) |
| 1. How would you rate the ability of clinical and translational researchers at your institution to access IT services and expertise? | - Very good - Good - Fair - Poor - My institution does not provide IT services and expertise for the clinical and translational research community |
| 1. How would you rate the ability of clinical and translational researchers at your institution to access BMI services and expertise? | - Very good - Good - Fair - Poor - My institution does not provide BMI services and expertise for the clinical and translational research community |
| 1. How well coordinated is IT and BMI leadership at your institution? | - Very good - Good - Fair - Poor - My institution does not provide BMI services and expertise for the clinical and translational research community |
| 1. Please describe the relationship of IT and BMI leaders at your institution. | - Integrated/Coordinated - Not Integrated/Coordinated - Other (please describe) |
| 1. How would you rate the funding of IT and BMI services for the clinical and translational research community at your institution? | - Very good - Good - Fair - Poor - Other (please describe) |
| 1. Does your institution currently have a BMI training program? | - Yes - No - Other (please describe) |
| 1. Does your institution currently have a department or other formal academic unit for BMI? | - Yes - No - Other (please describe) |
| 1. Does your institution currently have a Clinical and Translational Science Award (CTSA) and/or a Comprehensive Cancer Center (CCC) grant/contract? | - Yes - No - Other (please describe) |
